# A Ten-Stage Protocol for Assessing the Welfare of Individual Non-Captive Wild Animals: Free-Roaming Horses (*Equus Ferus Caballus*) as an Example

**DOI:** 10.3390/ani10010148

**Published:** 2020-01-16

**Authors:** Andrea M. Harvey, Ngaio J. Beausoleil, Daniel Ramp, David J. Mellor

**Affiliations:** 1 Centre for Compassionate Conservation, Faculty of Science, University of Technology, Sydney, NSW 2007, Australia; Daniel.Ramp@uts.edu.au; 2 Animal Welfare Science and Bioethics Centre, School of Veterinary Science, Massey University, Palmerston North 4474, New Zealand; N.J.Beausoleil@massey.ac.nz (N.J.B.); D.J.Mellor@massey.ac.nz (D.J.M.)

**Keywords:** Five Domains, welfare assessment, wildlife, free-roaming, wild, feral, horses

## Abstract

**Simple Summary:**

Vital for informing debates about the ways we interact with wild animals and their associated habitats is knowledge of their welfare status. To date, scientific assessments of the welfare of free-roaming wild animals during their normal day-to-day lives are not available, in part because the required methodology had not been developed. Accordingly, we have devised, and here describe, a ten-stage protocol for systematically and scientifically assessing the welfare of individual non-captive wild animals, using free-roaming horses as an example. Applying this ten-stage protocol will enable biologists to scientifically assess the welfare of wild animals and should lead to significant advances in the field of wild animal welfare.

**Abstract:**

Knowledge of the welfare status of wild animals is vital for informing debates about the ways in which we interact with wild animals and their habitats. Currently, there is no published information about how to scientifically assess the welfare of free-roaming wild animals during their normal day-to-day lives. Using free-roaming horses as an example, we describe a ten-stage protocol for systematically and scientifically assessing the welfare of individual non-captive wild animals. The protocol starts by emphasising the importance of readers having an understanding of animal welfare in a conservation context and also of the Five Domains Model for assessing welfare. It goes on to detail what species-specific information is required to assess welfare, how to identify measurable and observable indicators of animals’ physical states and how to identify which individuals are being assessed. Further, it addresses how to select appropriate methods for measuring/observing physical indicators of welfare, the scientific validation of these indicators and then the grading of animals’ welfare states, along with assigning a confidence score. Finally, grading future welfare risks and how these can guide management decisions is discussed. Applying this ten-stage protocol will enable biologists to scientifically assess the welfare of wild animals and should lead to significant advances in the field of wild animal welfare.

## 1. Introduction

There is a growing awareness of how human activities, including wildlife population management and rehabilitation, land management and other conservation activities, may influence the welfare of free-roaming animals in the wild [1,2,3,4,5,6,7,8]. Conservation and wildlife management practices have traditionally focused on assessing animal populations, using metrics like abundance, density and diversity; demographic parameters like sex ratios and age classes; and fitness metrics like survivorship and reproductive success. While valuable for some conservation purposes, such metrics provide little information about the welfare of individual animals within populations. However, survival does not necessarily imply good welfare since animals can survive despite persistently experiencing chronically unpleasant states [9,10,11,12]. Furthermore, the welfare of individual animals can influence the success of some conservation activities. For example, poor welfare may reduce fitness and reproductive success, and thus alter population trajectories. In addition, the public are increasingly aware of, and concerned about wild animal welfare [3,13]. Therefore, having knowledge of the welfare status of individual wild animals may contribute information directly relevant to ethical, legal and political debates about the ways in which we interact with wild animals and their associated habitats [14].

Methods for assessing welfare have been well developed for a range of captive animals [15,16,17,18,19,20,21,22,23], including for wild species [24,25,26,27,28]. Although a need to develop methodologies for assessing the welfare of free-roaming wildlife has been highlighted [1], to date, such assessments have been largely restricted to impacts of non-lethal or lethal control of unwanted species, such as rodents, possums, rabbits, kangaroos, camels, badgers, and horses [29,30,31,32,33,34,35,36,37,38,39,40,41,42,43,44,45,46,47]. Whilst a recent study explored some aspects of welfare in the daily lives of free-roaming wild dogs [8], protocols for purposefully, systematically and scientifically assessing the welfare of free-roaming wild animals undertaking their normal daily activities, remain elusive. Therefore, little is known about what positive and negative welfare impacts they might be experiencing and why. Moreover, robust scientific methods for capturing reliable and informative data to enable assessment of free-roaming wild animal welfare have not been well described.

In light of the above observations, we describe a ten-stage protocol designed to guide wildlife biologists and others who wish to apply a systematic, scientifically based approach for assessing the welfare of individual free-roaming wild animals. We use the term ‘free-roaming’ to distinguish between wild species that roam freely in a wild habitat from those that are in captivity (e.g., in a zoo or sanctuary). We use the term ‘wild’ to mean animals that are of a wild species or those that are non-domesticated (feral). We have avoided the term ‘feral’ since this is associated with negative public perceptions, and whether an animal is truly wild or feral has no influence on the principles of how its welfare is assessed. This protocol, based on the ‘Five Domains Model’ [48,49,50,51], will ensure that such assessments are as scientifically objective, systematic, structured, transparent and comprehensive as possible. Applying the protocol will also enable researchers to present a clear understanding of the limitations imposed on their particular assessment by the circumstances in which data collection and interpretation necessarily occur when studying wild free-roaming animals. We also suggest methods that may be employed to capture robust data to support such welfare assessments.

We illustrate the application of the ten-stage protocol by using wild free-roaming horses as an example. This species was chosen since the study is part of a broader project regarding the practical assessment of the welfare of wild free-roaming horses and implications for their management.

## 2. The Ten-Stage Protocol

1. Acquire an understanding of the principles of Conservation Welfare
2. Acquire an understanding of how the Five Domains Model is used to assess welfare status
3. Acquire species-specific knowledge relevant to each Domain of the Model
4. Develop a comprehensive list of potential measurable/observable indicators in each physical domain, distinguishing between welfare status and welfare alerting indices
5. Select a method or methods to reliably identify individual animals
6. Select methods for measuring/observing the potential welfare indices and evaluate which indices can be practically measured/observed in the specific context of the study
7. Apply the process of scientific validation for those indices that are able to be measured/observed, and insert validated welfare status indices into the Five Domains Model
8. Using the adjusted version of the Model that includes only the validated and practically measurable/observable welfare status indices, apply the Five Domains grading system for grading welfare compromise and enhancement within each Domain
9. Assign a confidence score to reflect the degree of certainty about the data on which welfare status has been graded
10. Including only the practically measurable/observable welfare alerting indices, apply the suggested system for grading future welfare risk within each Domain.

### 2.1. Stage 1: Acquire an Understanding of the Principles of Conservation Welfare

A new discipline of Conservation Welfare has recently been proposed to align traditional conservation approaches that historically focused on measures of ‘fitness’ (physical states), with more contemporary animal welfare science concepts which emphasise ‘feelings’ (mental experiences or affective states), that result from physical states. This enables a more holistic understanding of animals’ welfare states [52]. A common language and understanding relating to wild animal welfare are important starting points, since the way in which welfare is conceived influences the way it is evaluated and the emphases put on its different features [52]. The reader is referred to Beausoleil et al. 2018 [52] for a more detailed consideration of the value of seeking a shared welfare-related understanding between conservation scientists and animal welfare scientists under the heading of Conservation Welfare. 

Animal welfare is characterised mainly in terms of an animal’s mental experiences, in other words, how the animal may be experiencing its own life [52,53,54,55,56]. In animal welfare science, welfare is conceptualised as a property of individuals, belonging to species considered have the capacity for both pleasant (positive) and unpleasant (negative) mental experiences, a capacity known as sentience [52,57,58,59,60,61,62]. Contemporary animal welfare science aims to interpret indicators of biological function and behaviour in terms of the mental experiences that those indicators are likely to reflect [52]. Mental experiences, or affective states, are subjective and cannot be measured directly, but indirect indices can be used to cautiously infer affective experiences [48,49,50,51,52,63]. 

#### 2.1.1. Negative Affective States

There is a growing body of neurophysiological and behavioural evidence in non-human animals regarding the basis of negative affective states such as breathlessness, thirst, hunger, pain, fear, nausea/sickness, dizziness and weakness, and there are also validated links between measurable indicators of physical/functional states and some of these mental experiences [36,58,62,63,64,65,66,67,68,69,70,71]. For example, body condition is a measurable physical state that can be used as an indicator of hunger in some situations [72,73,74,75]. Likewise, certain behaviours can be used as indices of pain. For example, in horses, the combination of rolling, gazing and/or kicking at the abdomen along with inappetence may be interpreted as reflecting abdominal pain [76].

Some affective experiences are generated by the animal’s brain processing sensory inputs that register specific features of their internal physical/functional state. For example, water deprivation causes dehydration which leads to osmoreceptor-stimulated neural impulses passing to the brain generating the affective experience of thirst [67]. Thirst elicits the behaviours of seeking water and drinking, in order to correct dehydration, after which the mental experience of thirst ceases.

Other affective experiences may arise from externally stimulated sensory inputs that contribute to the animal’s perception of its external circumstances. For example, threatening situations such as the presence of predators or humans, separation from conspecifics, or environmental hazards such as fire, are registered via cognitive processing of sensory inputs from visual, auditory and/or olfactory receptors giving rise to anxiety and fear [52,64,66,67,69,71].

Whilst some negative experiences such as thirst and hunger motivate the animal to be behaviourally active in order to achieve resolution of the experience, others motivate the animal to reduce its activity. For example, weakness, sickness and pain often induce inactivity and seeking to be isolated from other animals [50]. These and other types of behaviour are referred to as ‘sickness’ behaviours and may facilitate recovery from disease and injury thereby enhancing survival [49,77]. Experiencing negative emotions to some degree is therefore essential in order to motivate life-sustaining behaviours, but it is the incidence, intensity and duration of these experiences that are important in determining the overall impacts on an animal’s welfare state. It is when negative experiences become extreme, prolonged or unavoidable, that an animal experiences the most severe compromises to its welfare [3,49,50].

#### 2.1.2. Positive Affective States 

Animals can also experience a range of positive affective states, and when experienced, these may enhance the animal’s welfare state [50,66,78,79,80,81]. Some positive mental experiences may occur as a result of behaviours that are directed at minimising negative affects [50]. For example, the smell, taste, textural and masticatory pleasures of eating a range of foods and the comfort of post-prandial satiety may occur with eating that is directed at relieving hunger [50,77,82,83]. Alternatively, other positive experiences may replace negative experiences when an animal is able to express more of its behavioural repertoire [50,51,55,78,79,80]. For example, foraging, affiliative social interactions, adolescent play behaviour, maternal behaviour and sexual activity are behaviours that infer positive mental experiences [50,55,64,69,83,84]. Despite living in stimulus-rich environments, expression of rewarding behaviours can be hindered in wild free-roaming animals. For example, in malnourished horses, more time and energy is spent searching for food. Hunger is also likely to dominate awareness and this, in turn, may reduce motivation to undertake rewarding behaviours [50,51]. Conversely, when food is plentiful, relief from the negative experience of intense hunger may re-motivate animals to utilise existing opportunities to engage in a range of rewarding behaviours [51]. Therefore, it is important to consider indicators of positive, as well as negative welfare states in wild free-roaming animals and to understand particular features of their ‘natural’ circumstances may compromise or enhance their welfare [85].

### 2.2. Stage 2: Acquire an Understanding of How the Five Domains Model Is Used to Assess Welfare Status 

**Figure 1:**
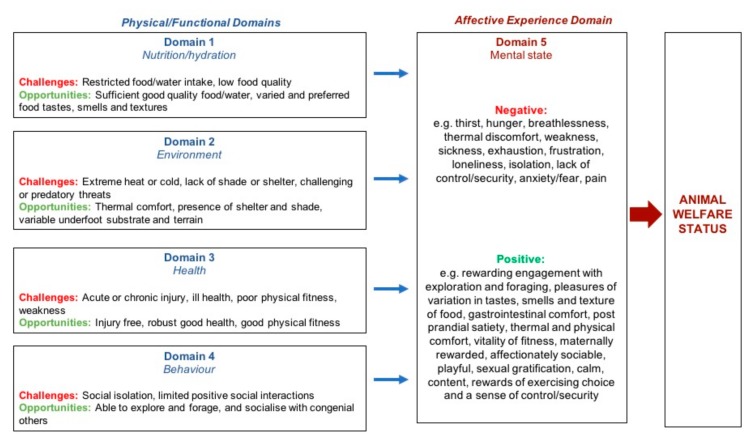
An abbreviated schema of the Five Domains Model (adapted from Littlewood and Mellor 2016 [86]), showing negative and positive physical/functional states or situations (Domains 1–4) and examples of their associated negative and positive mental experiences or affects (Domain 5), relevant to free-roaming wild horses. Taken together, these mental experiences represent the overall welfare state of the animal. A more detailed schema is available elsewhere [50].

The Five Domains Model [48,49,50,51] is consistent with, and structurally represents, the understanding that physical and mental states are linked (Figure 1). It is a device that facilitates systematic and structured welfare assessment of individual sentient animals, based on current understanding of the functional bases of negative and positive subjective experiences that animals may have [48,49,50,51]. Originally developed to assess welfare compromise in animals used in research, teaching and testing [48], it has since been broadened for use in companion animals, livestock, captive wild animals and animals designated as ‘pests’ [27,36,49,50,51,55,86,87,88,89,90]. 

The Five Domains Model comprises four interacting physical/functional domains of welfare; ‘nutrition’, ‘environment’, ‘health’ and ‘behaviour’, and a fifth domain of mental state (affective/mental experience) (Figure 1). The physical/functional domains focus on internal physiological and pathophysiological states (Domains 1–3) and external physical, biotic and social conditions that may alter the animals’ behavioural expressions (Domain 4) [49,50,51]. Following measurement of animal-based indices within each physical domain, the anticipated negative or positive affective consequences are cautiously assigned to Domain 5. It is these experiences that contribute to descriptions of the animal’s welfare state [49,50,51].

It is imperative that a sound understanding of the principles of Conservation Welfare (Stage 1) and the Five Domains Model (Stage 2) is gained prior to progressing to the next stages of the protocol.

### 2.3. Stage 3: Acquire Species-Specific Knowledge Relevant to Each Domain of the Model 

In order to appropriately apply the Five Domains Model to assess animal welfare, detailed species-specific knowledge is required. Table 1 illustrates the species-specific information within each of the four physical/functional domains, that is required to enable assessment of the welfare of free-roaming horses. Without a thorough understanding of what is normal for a species under optimal conditions, it is not possible to identify or interpret abnormalities. Acquiring species-specific knowledge will likely require extensive reading and advice from others having species-relevant practical experience, in addition to species-relevant nutritional, environmental, health and behavioural expertise. Accordingly, such holistic welfare assessments require multidisciplinary input [49,50,51].

All of the information required to make an informed assessment of the animal’s welfare status may not be available for the wild species of interest. However, systematically undertaking Stage 3 will help to identify knowledge gaps and related limitations in welfare assessments, thus guiding further research. 

### 2.4. Stage 4: Develop a Comprehensive List of Potential Measurable/Observable Indicators in Each Physical Domain, Distinguishing between Welfare Status and Welfare Alerting Indices

**Figure 2:**
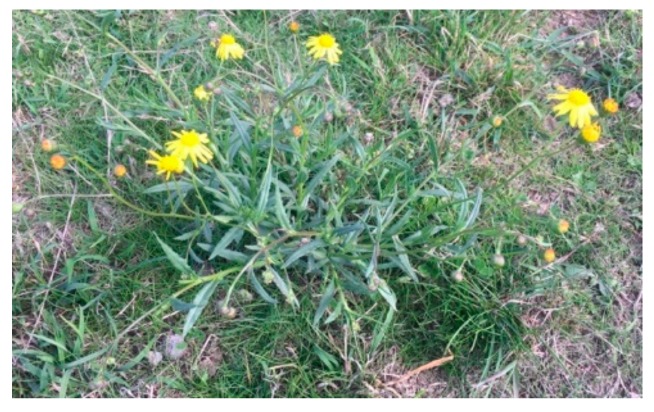
Ingestion of plants such as Fireweed (*Senecio madagascariensis*), can cause pyrrolizidine alkalosis in horses resulting in chronic liver failure and eventual clinical signs of diarrhoea, weight loss, subcutaneous oedema, neurological disease and ultimately death [91]. Observing an abundance of these plants within a wild horse’s habitat should act as a welfare alerting factor to prompt further monitoring of horses for presence of these clinical signs, and/or to consider this as a potential cause of any unexplained mortalities. Image A.M. Harvey.

Based on knowledge of the theory of animal welfare and its importance in a conservation context (Stage 1 and 2), and on species-specific knowledge (Stage 3), the next stage is to develop a list of potential indicators of various physical and thus affective states (both positive and negative) that the animals might experience. Measurable or observable indicators can be animal-based, such as body condition score and behaviour, or resource-based, such as forage quality and weather conditions (Table 2). Some indices (specifically animal-based indices) will be direct indicators of physical states, and therefore reflect aspects of welfare status. Others will be indicators of the risk of particular states occurring, or welfare alerting indices (all resource-based indicators and some animal-based indicators). Welfare alerting indices do not directly reflect the animal’s current welfare state, but they can direct attention in future assessment towards specific animal-based indices (e.g., Figure 2). All assessments are made on individuals, but some resource-based indicators may apply to a number of individuals and therefore have group applications. 

#### 2.4.1. Search for Previously Described Indices

Literature searches should be performed to develop a list of potential indices that may have already been described for use in welfare assessments of the species of interest, either in a free-roaming context or in a domesticated/captive context, and to evaluate their suitability. For example, various horse welfare assessments have been described and some of the indices used may be practical to apply to wild free-roaming horses [16,19,20,22,23,92,93]. Published information may also exist with regard to methods for measuring or observing some of these indices. For example, in horses there are well described protocols for assessing body condition score [94,95], and behavioural [76] and facial [96] signs of pain have been described, with development of a horse grimace scale for assessing some types of pain [97]. 

#### 2.4.2. Some Animal-Based Indices Provide Welfare Status Information

**Figure 3:**
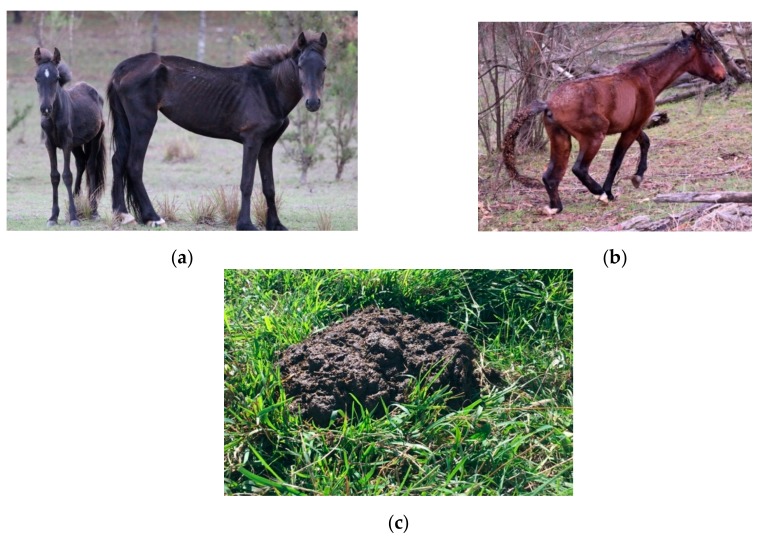
Examples of externally observable animal-based indices that provide information about welfare status. (**a**) An emaciated mare with a body condition score of 1/9 [93] with a stunted yearling; (**b**) A horse with a left hind lower limb injury; (**c**) Diarrhoeic faeces, indicative of gastrointestinal disease such as parasitism. Images A.M. Harvey.

**Figure 4:**
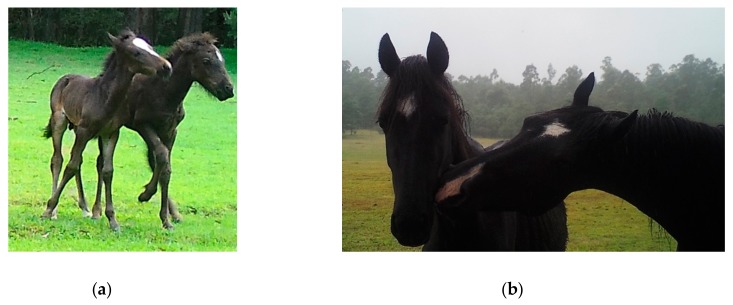
Examples of qualitative assessment of behaviour as an externally observable animal-based indicator of positive mental experiences associated with: (**a**) Play, and; (**b**) Affectionate sociability. Images A.M. Harvey.

Only animal-based indices can contribute information to the assessment of overall welfare status, since they provide the most direct evidence of what the animal may be experiencing [48,49,50,51,98]. Animal-based indices may be externally observable, or internally measurable, as illustrated in Table 3. Externally observable indices can provide easily observable evidence of welfare compromises in each domain, and are the most practical indices to use in free-roaming animals (Figure 3, Table 3). Quantitative measures of behaviour, such as time budgets, have been most commonly applied by wildlife biologists [99,100,101,102]. However, since behaviour reflects a complex level of functioning, qualitative assessment can also inform assessment of the animals’ affective state and whether positive or negative mental experiences are occurring [103,104,105,106] (Figure 4). To date, qualitative behavioural assessments do not appear to have been scientifically studied in free-roaming wild animals, and it is important that the context of the behaviour is considered carefully when making such assessments [107].

Internally measurable indices relate to physiological, pathological or clinical conditions (Table 3). These indices are not routinely used for day-to-day welfare assessments, and are problematic to measure in free-roaming wild animals. Some indices such as cortisol and reproductive hormones can be measured in faeces, which makes this more feasible for use in wild animals. However, interpretation of many of these indices is not straightforward. For example, while faecal [108,109,110] and hair [111,112] cortisol concentrations have been employed as a physiological index of stress [108,109,110,111,112], the significance of non-specific stress for an animal’s mental experience is unclear [52,113]. Cortisol and many other physiological parameters are non-specific and do not indicate if the experience was positive (e.g., excitement, arousal) or negative (e.g., pain, fear, hunger). Further, lack of elevated cortisol concentrations does not mean that the animal is not experiencing something unpleasant. Cortisol concentrations are also affected by many other variables (e.g., species, sex, reproductive status, circadian rhythms), further hindering interpretation [52]. Accordingly, an absence of detailed contextual information limits how informative cortisol measurements are in wild free-roaming animals.

#### 2.4.3. Some Animal-Based Indices Provide Welfare Alerting Information

**Figure 5:**
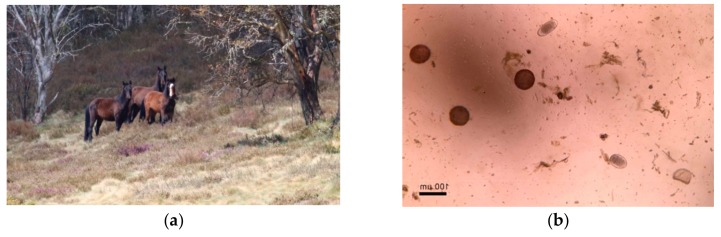
(**a**) Low reproductive success, i.e., an absence of foals, particularly when combined with observing poor forage availability, may be an alerting indicator for chronic malnutrition, since poor nutrition in horses is known to have negative impacts on their fertility [93]; (**b**) Presence of parasite eggs in the faeces can be used as an indicator of the presence of certain gastrointestinal parasites [118]. However, faecal egg counts (FEC) give no indication of the severity of any associated pathology and cannot be used directly to make inferences about the animals’ mental experience. FECs therefore are welfare alerting indices, with a high FEC raising awareness that gastrointestinal pathology and subsequent clinical signs (e.g., diarrhoea, abdominal pain) may be more likely to arise in the future. Images A.M. Harvey.

Animal-based indices traditionally collected by wildlife biologists (e.g., population dynamics, home range features and size, reproductive rates and survival rates), may not directly reflect the mental experiences of individuals, however, they may provide relevant contextual information. For example, low reproductive success, smaller herd sizes and/or larger home ranges, may reflect physiological states (e.g., chronic malnutrition) that would generate negative affective states of relevance to welfare [94,114,115,116,117] (Figure 5a). Consequently, such indices may provide information about future welfare risks, and thus become important welfare alerting indices. Some other animal-based indices, such as faecal egg counts (FECs), may also only provide welfare alerting rather than welfare status information (Figure 5b, Table 2), because, when FEC is high, free-roaming horses frequently do not exhibit overt clinical signs of disease [118,119]. Hence, interpreted in isolation they do not necessarily indicate presence of intestinal pathology and any related negative experience.

#### 2.4.4. Some Animal-Based Indices Can Be Interpreted in Combination with Resource-Based Indices

**Figure 6:**
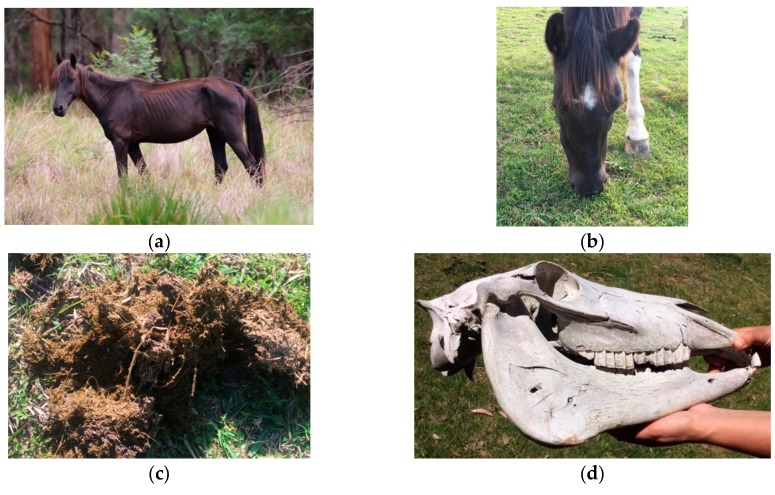
(**a**) Dental disease may be suspected as one possible cause of low body condition. For example, if an individual horse is in poor body condition when feed is plentiful, conspecifics are in good body condition, and there is no obvious alternative reason for the individual to be in poor condition (e.g., not lactating or injured); (**b**) Quidding (dropping food from the mouth whilst chewing) and/or food pouching in the mouth lateral to the cheek teeth (as shown on the horse’s right cheek in the photograph) are associated with pain from dental disease; (**c**) Long unchewed grass fibres in the faeces are suggestive of reduced chewing ability with dental disease [120,121]; (**d**) Information on the incidence of dental disease in a population as a whole (i.e., alerting information) may be provided by examination of the dentition of skulls found in the horses’ habitat. Images A.M. Harvey.

In some situations a combination of resource-based and animal-based indices may provide indirect relevant information about current welfare status and future risk. For example, dental disease can be an important cause of both morbidity (e.g., pain, malnutrition) and eventual mortality (malnutrition) in horses [120,121], and several externally observable indices can be suggestive of clinically significant dental disease (Figure 6).

### 2.5. Stage 5: Select a Method or Methods to Reliably Identify Individual Animals

In order to assess animal welfare at an individual level, individuals need to be identifiable. Non-interventional identification methods may be suitable for some species. For example, in horses a combination of coat colour and natural markings may be used [122,123,124]. Where such approaches are not possible, alternative methods may be required, such as marking with paints or dyes, or applying tags [125]. Factors such as distance from the animal during observations and visibility are important considerations in choice of identification method. Animal welfare impacts associated with capture/handling/restraint, application of any marks/tags, wearing of the mark, and impacts of observations should be assessed. The welfare impacts of different methods of marking have been previously reviewed, and should be considered along with other advantages and disadvantages of the marking method, before deciding upon those most appropriate for identification [125,126,127,128,129,130].

### 2.6. Stage 6: Select Methods for Measuring/Observing the Potential Welfare Indices and Evaluate Which Indices Can Be Practically Measured/Observed in the Specific Context of the Study 

Having decided, based on species-specific knowledge (Stage 3), what resource-based and animal-based indices are important for assessing welfare in the species of concern (Stage 4), and how individual animals are going to be identified (Stage 5), the methods of practically measuring/observing the required indices then need to be considered.

Collecting information on the welfare of wild free-ranging individual animals is logistically challenging: their habitats may be difficult to access; the animals may be difficult to observe because of natural features such as vegetation and topography, in addition to fear of humans, and they may be unobservable for significant periods or at repeated intervals. In some situations it may also be challenging to locate the individuals that may be experiencing the worst welfare impacts, as they may hide, be less mobile, more distant from conspecifics and in habitats/terrain that make visualising them difficult.

**Figure 7:**
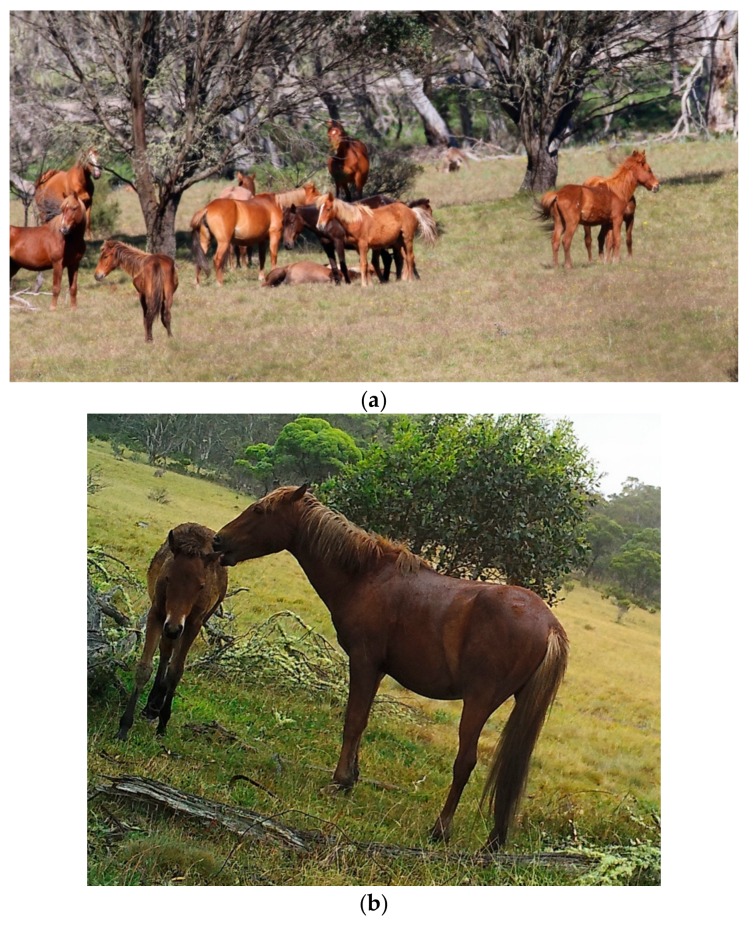
Where possible, combining methods can provide the most comprehensive information. For example: (**a**) Is a photograph taken from direct distance observation showing herd size, structure and social interactions. Direct observations, where possible, also provide a wealth of behavioural information, whereas; (**b**) Is a closeup camera trap image. This can enable easier measurement/observation of animal-based indices such as body condition score, coat condition, hoof condition, presence of injuries and presence of food pouching. Images A.M. Harvey.

Historically, data on free-roaming animals have been obtained using methods such as direct observations (e.g., herd size, behaviour, body condition score), trapping (e.g., sex, weight, size) and GPS collaring (e.g., home range, distance travelled) [99,100,101,102,122,123,124,125,126,127,128,129,130,131]. Although these methods can yield useful information, they themselves often have significant welfare implications [125,126,127,128,129,130], provide a very narrow range of data, and there may be bias of the individuals sampled (e.g., direct observation is likely biased to those individuals within habitats where direct visualisation is possible). With more recent advances in technologies, it is now possible to obtain a wider range of information about free-roaming animals, and for longer periods of time, using techniques such as camera traps and drones [132,133,134,135,136,137] (Table 4). Advantages and limitations of each potential method need to be considered for the species and context of the research, and the highest yielding methods may vary. For example, for free-roaming horses residing on open grassland or desert habitat, direct observations or drones may be the most effective way to obtain animal-based data. In contrast, in a woodland habitat, where trees may interfere with direct visualisation of animals, camera traps may be more appropriate. Combined, these methods can provide complementary information (Figure 7).

In some situations, direct animal-based indices may be impractical, but there could be alternative indices that indirectly provide relevant information. For example, it is not practical to assess the dentition of free-roaming wild horses, but some indices can be observed that are indirectly suggestive of clinically significant dental disease (Figure 5).

Methods should be evaluated by undertaking pilot studies to identify which of the potential indices are practically feasible to measure/observe in the context of the study. Indices that are not practically able to be measured/observed with currently available methods should be archived. This enables them to be considered at a later stage when evaluating the limitations of the welfare assessments (Stage 9), and to be revisited when future technological advances may make them more feasible to measure or observe.

### 2.7. Stage 7: Apply the Process of Scientific Validation for Those Indices that Are Able To Be Measured/Observed, and Insert Validated Welfare Status Indices into the Five Domains Model

Once it has been established which indices can be practically measured/observed in the species and context of interest (Stage 6), these indices then need to be scientifically validated. Ideally, validation of welfare indices requires prior demonstration of the relationship between an observed indicator and the physical/functional impact (Domains 1–4), and of the relationship between the physical/functional impact (Domains 1–4) and the inferred mental experience (Domain 5). These steps of scientific validation have been described in detail elsewhere [63]. For example, detection of raised plasma osmolarity by osmoreceptors increases water-seeking and drinking behaviour, and drinking eliminates water-seeking behaviour [67], validating the link between the externally observable indicator of water-seeking behaviour/drinking, and the internally measurable indicator of dehydration, plasma osmolarity. Affective neuroscience provides evidence of the link between the physical state of dehydration (increased plasma osmolarity) and the mental experience of thirst, via neurohormonal pathways transmitting afferent inputs from osmoreceptors to higher brain centres associated with emotions [67]. 

Ideally, evidence of these relationships should relate to the species and context of interest, but where this is not available, evidence from the same species in a different context (e.g., in captivity), or a similar species, can be cautiously extrapolated. In many situations, the complete body of evidence to achieve such validation is not available and the level of confidence in the validation of indices should be indicated [63].

Thus, this process will also highlight further knowledge gaps, and what further evidence may be required to strengthen the confidence between the suggested animal-based indices and inferred mental experiences.

In some cases, a direct animal-based indicator may not be practical to measure/observe in free-roaming animals, but there may be scientific evidence to support the use of an indirect indicator, which may be resource-based. For example, in free-roaming animals, water seeking or drinking behaviours can be difficult to observe. Therefore, thirst may be indirectly judged based on the resource-based indices of how available water sources are in relation to required frequency of drinking, based on the best available data for the species of interest. In the absence of direct measures, strength of motivation to drink could also be assessed by the distance the animal is willing to travel to reach a water source. Factors other than location of water sources would also need to be considered since impaired water access may occur for other reasons, such as illness or injury.

Indices that cannot be scientifically validated as indicators of the animals’ mental experience (e.g., poor hoof condition in the absence of an abnormal gait), should be archived for consideration in future validation studies. Some of these archived indices may still provide valuable alerting information. All welfare alerting indices (Table 3) should be evaluated and graded separately from welfare status indices, as described in Stage 10. 

### 2.8. Stage 8: Using the Adjusted Version of the Model that Includes Only the Validated and Practically Measurable/Observable Welfare Status Indices, Apply the Five Domains Grading System for Grading Welfare Compromise and Enhancement Within Each Domain

Once the indices that can be practically measured/observed (Stage 5), which are deemed to be sufficiently validated (Stage 7), have been inserted into the Five Domains Model, the next stage is to apply the grading system.

In order to standardise the assessment of animal welfare across different individuals and/or different assessors, and to monitor animal welfare over time, a reliable, repeatable and practical method of grading is required. Grading welfare compromise and welfare enhancement, and the operational details of the Five Domains Model have been previously described [50,51,87,88]. It should be noted that such grading does not necessarily provide a comprehensive assessment of welfare status; rather it provides an assessment of those indices of welfare that can be assessed and interpreted in terms of the mental experience they are associated with, in the particular species and context of interest. In the case of free-roaming animals the range of welfare-relevant indices that can be assessed will usually be more limited than that for animals in captivity. 

Grading the impact of mental experiences on welfare status involves a different approach depending on whether the experiences are negative (welfare compromise) or positive (welfare enhancement) [50,51,87] (Table 5).

#### 2.8.1. Grading Welfare Compromise (Negative Mental Experiences)

The grading system applies a five-tier scale (A–E) to each of the Five Domains, representing increasingly severe impacts, ranging from none to very severe (Table 6) [50,51,87]. Information from the scientifically validated measurable/observable indices decided upon in Stage 7 is used to assign the grade of physical impact (A–E) in the first 4 domains. Knowledge of the association between those physical impacts and the associated mental experiences is used to infer the type of unpleasant experiences in Domain 5. The grades assigned in Domains 1–4 are used to infer the severity and duration of those experiences in Domain 5. The grade assigned in Domain 5 is usually the same as the highest of the grades in Domains 1–4, to reflect the most severe negative mental experience. This grade is the overall welfare compromise grade (Table 6).

There may, however, be insufficient information to define impacts with the degree of precision implied by a five-tier scale, and in this case the grading matrix can also be adapted to a simpler three-tier scale to represent ‘no to low’, ‘moderate’, and ‘severe’ compromise [51] (Table 7). 

#### 2.8.2. Grading Welfare Enhancement (Positive Mental Experiences) 

**Figure 8:**
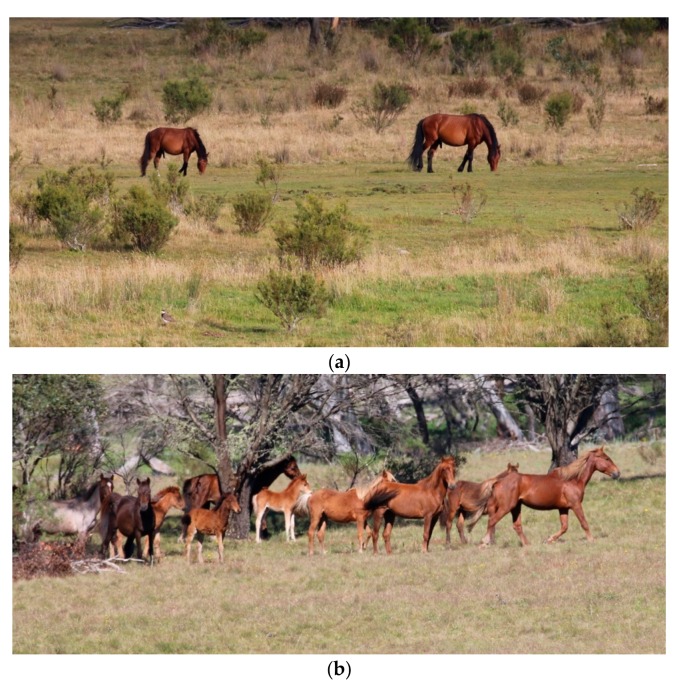
The images of the two groups of horses in (**a**) and (**b**) were taken from a large population of horses, but due to the social organisation of herds, illustrate the difference in the ability of some horses to engage in affectionate social interactions, maternal, sexual and play behaviour much more than others: (**a**) These horses, 2 bachelor stallions, would be graded as ‘+’ for welfare enhancement associated with opportunities in Domain 4, whereas; (**b**) These horses, being in a large mixed age/sex herd with multiple foals, would be graded as ‘+++’ for welfare enhancement associated with such opportunities in Domain 4. Images A.M. Harvey.

The described grading system applies a four-tier scale (0, +, ++, +++), representing ‘no’, ‘low-level’, ‘medium-level’ and ‘high-level’ enhancement [50,51,87], but as above could also be simplified to a two- or three-tier scale when information relating to positive mental experiences is sparse. Grading of welfare enhancement has three elements; (i) the availability of *opportunities* for the animal to engage in self-motivated rewarding behaviours, (ii) the animals actual *utilisation* of those opportunities, (iii) making a cautious judgement of the degree of *‘positive affective engagement*’. For example, in free-roaming horses, when grading positive mental experiences (Domain 5) associated with impacts in Domain 4 (behaviour), opportunities for horses to engage in free movement, exploration, foraging a range of vegetation of varying tastes and textures, to have affectionate social interactions with bonded conspecifics and engage in maternal, sexual or play behaviour, would be expected. However, for a variety of reasons, a horse may not be able to utilise these opportunities, and consequently will not exhibit behaviours that would provide evidence of positive mental experiences. This may occur where there is welfare compromise. For example, malnutrition, dehydration, hypothermia, injury and illness may all impair an animal’s ability to engage in activities that may otherwise be pleasurable [36,50,70,71]. The ability to engage in positive social interactions may also be impacted by aspects of social organisation and group composition [139] (Figure 8). Table 5 illustrates one way in which the interaction between compromise and enhancement has been conceptualised, i.e., severe compromise hinders enhancement.

The use of numerical scores in the grading system is explicitly rejected in order to avoid scientifically unjustified aggregation of scores and to avoid implying a degree of precision that is not achievable when qualitatively assessing subjective affective states [48,49,50,51]. Scientifically informed best judgement is an important aspect of grading with the Five Domains Model, and so the grading scheme should act as a guide only, but be utilised alongside informed interpretation [50,51]. Detailed examples of species and situational specific grading matrixes and application of this grading system can be found elsewhere [50,51,87,88]. 

### 2.9. Stage 9: Assign a Confidence Score to Reflect the Degree of Certainty about the Data on Which Welfare Status Has Been Graded

When the grading system is applied to assess individual animal welfare (Stage 8), a confidence score should then be assigned to the overall welfare status grade, to reflect the degree of certainty about the data upon which the grade was based [88]. We recommend a three-tier scoring system where L = low confidence, M = moderate confidence and H = high confidence.

The confidence score should reflect the knowledge gaps and limitations of the assessment, including gaps in species-specific knowledge (Stage 3), any challenges with individual animal identification (Stage 5) and the archived indices that could either not be practically measured/observed with currently available methods (Stages 6), or which could not be sufficiently validated (Stage 7). These are critical actions both for directing further research to improve future welfare assessments, and for informing the level of confidence with which individual welfare can currently be assessed in the species and context of interest.

In addition, a range of other factors should be considered including: whether all indices in the grading scheme could be measured/observed in the individual being assessed; the number of and/or duration of observations of the animal; whether indices were measured/observed from several methods combined or a single method; the implications if all methods could not be applied (e.g., still images only vs. video recordings vs. direct observations); and the distance of the assessor from the animal/image/video recordings when measurements/observations were made. The importance of some of these factors may also vary depending on the degree of welfare compromise. For example, if a welfare compromise status grade of E is assigned to a horse with a body condition score of 1/9, or a horse with a broken leg, the confidence in that score may be high despite the possibility that the grade was based on data from a single still image of the horse. In contrast, if a welfare status grade of A was assigned to a horse based on a single still image, the confidence in that score would likely be low.

### 2.10. Stage 10: Including Only the Practically Measurable/Observable Welfare Alerting Indices, Apply the Suggested System for Grading Future Welfare Risk within Each Domain

**Figure 9:**
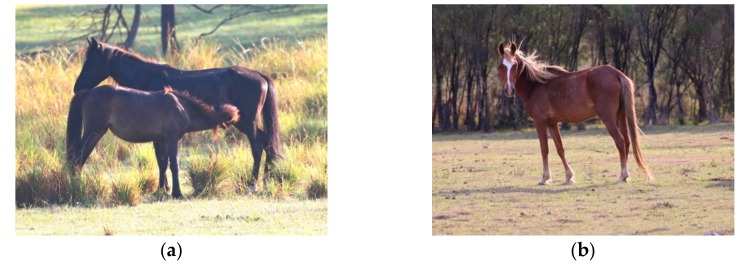
These images illustrate the value in grading welfare alerting indices. Both of these mares have the same grading for physical impacts in Domain 1 based on the animal-based measure of a body condition score of 3/9. However, alerting indices suggest that: (**a**) This mare has a low risk of further welfare compromise (and high likelihood of future improvement). This is because forage availability is good, it is the end of winter so forage quality and availability are likely to improve, and her yearling foal will soon be weaned, reducing nutritional demands on the mare. Accordingly, immediate intervention is not required, but body condition and forage availability should preferably be reassessed after another 6–12 weeks as intervention may be required if there was no improvement in body condition; (**b**) Conversely, this non-lactating mare has a high risk of further welfare compromise of increasing severity. This is because forage availability is poor and unlikely to improve because it is the end of Spring, and the mare is already in poor body condition despite the absence of additional nutritional demands from nursing a foal. In this case, therefore, the recommendation may be for immediate intervention or closer monitoring with intervention if her body condition were to decreased below 3/9 within the following month. Images A.M. Harvey.

From the comprehensive list of potential welfare alerting indices (Stage 4), select only those that can be measured/observed and interpreted (Stage 6). Some of these may be animal-based measures that were not able to be scientifically validated as indicators of mental experiences (Stage 7). Assessing such alerting indices separately from assessment of welfare status (Stage 8), can draw attention to risks of future welfare compromises and to what, if any, actions may be taken to mitigate these risks (e.g., Figure 9). This is particularly relevant to the situation of free-roaming animals, as unlike animals in captivity, immediate action based on a single welfare assessment or routine frequent monitoring of welfare may be impractical.

We therefore propose the use of an additional three-tiered scale for the overall grading of welfare alerting indices, representing ‘no to low’, ‘moderate’ and ‘high’ risk of further welfare compromise of increasing severity (Figure 9). Welfare alerting indices interpreted in combination with welfare status (Stage 8), should enable recommendations to be made relating to: (i) whether any immediate intervention is required, or (ii) whether further assessment or ongoing monitoring should be implemented, and what form that should take and (iii) the point at which intervention would be required to ameliorate increasing welfare compromise, where the risk of further compromise occurring is high.

## 3. Concluding Remarks

The ten-stage protocol described here illustrates how the well-established Five Domains Model can be systematically applied to assess the welfare of individual free-roaming wild animals. This paper therefore forms a template for making such welfare assessments in free-roaming wild terrestrial species by applying the principles outlined here.

Applying the Model to such animals will help to identify previously unrecognised features of poor and good welfare by more precisely characterising scientifically validated negative and positive mental experiences, and their evaluation, as opposed to the commonly used imprecise and non-specific descriptors such as ‘suffering’ and ‘stress’ [6]. Utilising qualitative grading allows the monitoring of the welfare status of animals in different circumstances and at different times, thus providing scientifically informed and evidence-based guidance for decisions to intervene or not, in addition to enabling assessment of responses to any interventions that are implemented.

Nevertheless, it is important to recognise the limitations of the Model and its use in the assessment of wild animal welfare. Only specific indices and mental experiences that can be identified and interpreted can be assessed; there will be variable levels of confidence with which particular experiences may be inferred to be present in different circumstances, and differing precision with which each mental experience may be graded, as well as an inability to determine relative impacts of those different experiences on welfare status [51]. For some species, in some contexts, it may become evident that very few welfare indices can be assessed and interpreted, significantly hindering welfare assessments. However, this then highlights and identifies the knowledge gaps that need to be filled. As such, it provides a sound foundation for further research into the welfare of wild free-roaming animals.

## Figures and Tables

**Table 1:** Illustration of the species-specific information required to assess welfare of free-roaming horses.

Domain	Species-Specific Information Required
**1: Nutrition**	Water requirements: volume, frequency, preferred water sources, factors influencing water requirements, adaptations to and impacts of water restriction
	Nutritional requirements and preferences
	Common nutritional deficiencies and excesses and their causes, plant toxicities
	Assessing body condition, body condition scoring systems, optimal body condition score, factors affecting body condition
**2: Environment**	Habitat preferences, and factors affecting habitat selection and use
	Preferred underfoot substrate and terrain
	Thermoneutral zone, impacts of extreme climate events, signs of thermal stress
**3: Health**	Common non-infectious diseases and their clinical signs, risk factors, aetiology, diagnosis and prognosis
	Common infectious diseases and their clinical signs, epidemiology, mode of infection, characteristics of infectious agent (e.g., life cycle, survival in environment, involvement of other species)
	Common injuries and their clinical signs, risk factors, aetiology, diagnosis and prognosis
	Sickness and pain behaviours
**4: Behaviour**	Social organisation and factors affecting it
	Population dynamics
	Reproductive physiology and behaviours; oestrous, courtship, mating, gestation, parturition, lactation, maternal and newborn behaviour
	Normal range of behaviours and time budgets
	Social behaviour (including ‘rewarding behaviours’ e.g., play, allogrooming and other positive affiliative behaviours) and communication

**Table 2:** Examples of animal-based and resource-based indices that may be measured or observed in free-roaming horses and which measures directly reflect mental experiences, i.e., welfare status, compared to welfare alerting indices that reflect welfare risk.

Domain	Animal-Based Indices		Resource-Based Indices All Alerting
	Index	Status/Alerting	
1: Nutrition	Body condition score	Welfare status	Water availability/sources (e.g., number, accessibility, reliability, proximity to core home range)
	Reproductive status (e.g., mature lactating female)	Welfare alerting	Predominant vegetation type in home range Mineral analysis of vegetation
			Grass quality and length
			Competition for resources
2: Environment	Spatial and temporal use of habitat Shivering Profuse sweating	Welfare alerting Welfare status	Weather (e.g., temperature, humidity, direct sun exposure, wind, rainfall, extreme weather conditions such as snow, hail, fire) Habitat (e.g., presence of and nature of protection from wind/sun/rain i.e., shelter and shade), terrain, substrate, ability to disperse to other habitats Anthropogenic activities (e.g., presence of roads, fencing, habitat destruction, use of habitat for other activities, noise
3: Health	Coat, skin, hoof condition Lameness Visible injuries, other visible physical abnormalities	Welfare status	Environmental conditions that may predispose to certain health conditions (e.g., heavy rain, moist substrates)
	General demeanour, mobility, gait, posture		Hazards that may predispose to injury (e.g., fencing, roads, terrain)
	Sickness behaviours		Presence and abundance of toxic plants
	Faecal quality		
	Dentition of any skulls found (e.g., dental pathology and age at death)	Welfare alerting	
	Faecal egg counts, *Strongylus vulgaris* molecular diagnostics (PCR)	Welfare alerting	
4: Behaviour	Quantitative (e.g., time-budget behaviours, frequency/duration of positive affiliative interactions) and qualitative (e.g., alert, relaxed, weak) assessment of behaviours	Welfare status	Opportunities to express complete range of normal behaviours; affected by environment and conspecifics
	Population dynamics and social organisation	Welfare alerting	

**Table 3:** Examples of animal-based indices that may provide information about welfare status.

Externally Observable Indices	Internally Measurable Indices
Growth rates and achievement of developmental milestones in young animals	Measurement of heart rate and core body temperature
Reproductive success	Measurement of various blood parameters such as complete blood count and serum biochemistry
Body weight and/or body condition score	
Presence of injuries, wounds, lameness, diarrhoea, nasal discharge, food pouching, quidding	Measurement of cortisol and reproductive hormones in urine, faeces and hair
Coat condition and presence of skin lesions	
Social behaviours, sickness or pain behaviours	Faecal egg counts

**Table 4:** Summary of methods that may provide information relevant for the welfare assessment of free-roaming wild horses.

Method	Relevant Information
Assessment of maps	Identification of cleared vs. forested areas and size of different habitats, geographical limitations to dispersal and recruitment, steepness of terrain, location of roads, rivers/creeks
Geographical and meteorological data	Temperatures, rainfall, snow, wind, known environmental information, vegetation types, etc.
Ground surveys	Essential for verifying information from maps, identifying presence of water in creeks, access to water sources, type of vegetation and abundance of food, direct visualization of the species of interest, and other species sharing the same habitat, evaluating presence/distribution of faeces, identifying good camera trap sites
Direct observations with or without photographs and/or videos	Best for evaluating behaviour and identifying herd composition but the variable distance between observer and horse can be more limiting in accurate body condition scoring, assessing hooves, skin lesions etc. Importantly many horses cannot be directly observed
Camera trap individual still images	Identifying individuals, sex, coat condition, skin lesions, hoof condition, body condition, limited behavioural information such as social interactions but not possible to identify sickness behaviours and gait abnormalities
Camera trap group still images	Herd compositions and sizes, foaling rate, approximate home ranges, mortality rate
Camera trap video images	Gait, demeanour, presence of lameness, weakness, occurrence of quidding/food pouching, play behaviour, positive affiliative interactions
Drones	Herd compositions, foaling rate, body condition, behaviour
Collection of faecal samples	Faecal consistency and colour, faecal egg counts, specific parasite molecular diagnostics, faecal cortisol and other hormone assays [108,109,110,138]

**Table 5:** A conceptual matrix of combined grading of welfare compromise and welfare enhancement (adapted from Mellor and Beausoleil 2015 [50]).

Welfare Compromise Grade	Welfare Enhancement Grade			
	None (0)	Low Level (+)	Med Level (++)	High Level (+++)
A None	-	A/+	A/++	A/+++
B Low	B/0	B/+	B/++	-
C Mild to moderate	C/0	C/+	-	-
D Marked to severe	D/0	-	-	-
E Very severe	E/0	-	-	-

**Table 6:** An example of grading welfare compromise in a horse with a lower limb injury resulting in severe lameness ^1^.

Domain of Potential Welfare Compromise					
1 Nutrition	2 Environment	3 Health	4 Behaviour	5 Mental Status	Overall Welfare Compromise Grade
C	B	D	C	D	D

^1^ Based on the severity of the injury and associated debility, a D grade has been assigned in Domain 3 (Health). The lameness has been observed to moderately impact on behaviour (inability to keep up and interact with the rest of the herd), leading to C grade in Domain 4 (Behaviour). Observations of reduced ability to forage and graze and a body condition score of 3/9, led to a C grade in Domain 1 (Nutrition). The horse’s environment is unchanged and the horse has easy access to shade and shelter, however the steep terrain is more challenging for the injured horse to negotiate, leading to a B grade in Domain 2 (Environment). The inferred mental experiences from these physical states include pain, hunger, and likely exhaustion, and possibly frustration and isolation. These are integrated to assign a grade in Domain 5 (Mental status). As the pain associated with the degree of lameness is considered to be severe, and of chronic duration, grade D has been assigned to Domain 5. This is the overall welfare compromise grade.

**Table 7:** An example of a modified three-tier grading system for assessing physical impacts in free-roaming horses within Domain 1 and associated negative experiences in Domain 5.

Measurable/Observable Indices	Compromise Grade		
	No to Low	Moderate	Severe
Access to water	Able to access water at least every 6–12 h. May be up to 12 h interruption in water supply in cool weather	Able to access water every 12–24 h. May be up to 12 h interruption in water supply in hot weather and up to 24 h interruption in water supply in cool weather. Interruption to water supply may be due to distance to water, or difficulty accessing water due to reduced mobility or competition with conspecifics for limited water supply	Unable to access water within 48 h in cool weather or 24 h in hot weather. Water not available, water sources blocked/dried out in drought or injury/illness preventing ability to access water
*Domain 5 Negative affective experience inferred: Thirst*	*No to very low-level thirst*	*Moderate thirst*	*Severe thirst*
Body condition score (BCS) and food availability	Optimal body condition (5–6/9) with good grass coverage within home range	Moderately thin (4/9) to thin (3/9) body condition with poor grass coverage within home range	Very thin (2–3/9) to emaciated body condition (1/9 or less) with very poor grass coverage within home range
*Domain 5 Negative affective experience inferred: Hunger*	*No to very low-level hunger*	*Moderate hunger*	*Severe hunger, weakness*

## References

[1] Kirkwood J.K., Sainsbury A.W., Bennett P.M. (1994). The welfare of free-living wild animals: Methods of assessment. Anim. Welf..

[2] Finn H.C., Stephens N.S. (2017). The invisible harm: Land clearing is an issue of animal welfare. Wild. Res..

[3] Beausoleil N.J., Appleby M.C., Weary D.M., Sandoe P. (2014). Balancing the need for conservation and the welfare of individual animals. Dilemmas in Animal Welfare.

[4] Ramp D., Bekoff M. (2015). Compassion as a Practical and Evolved Ethic for Conservation. BioScience.

[5] Dubois S., Fenwick N., Ryan E.A., Baker L., Baker S.E., Beausoleil N.J., Carter S., Cartwright B., Costa F., Draper C. (2017). International consensus principles for ethical wildlife control. Conserv. Biol..

[6] Wallach A.D., Bekoff M., Batavia C., Nelson M.P., Ramp D. (2018). Summoning compassion to address the challenges of conservation. Conserv. Biol..

[7] Hampton J.O., Hyndman T.H. (2019). Underaddressed animal-welfare issues in conservation. Conserv. Biol..

[8] Fraser-Celin V.-L., Hovorka A.J. (2019). Compassionate Conservation: Exploring the Lives of African Wild Dogs (*Lycaon pictus*) in Botswana. Animals.

[9] Main D.C.J., Whay H.R., Green L.E., Webster A.J.F. (2003). Effect of the RSPCA freedom food scheme on welfare of dairy cattle. Vet. Rec..

[10] Whay H.R., Main D.C.J., Green L.E., Webster A.J.F. (2003). An animal-based welfare assessment of group-housed calves on UK dairy farms. Anim. Welf..

[11] Korte S.M., Olivier B., Koolhaas J.M. (2007). A new animal welfare concept based on allostasis. Physiol. Behav..

[12] Walker M.D., Duggan G., Roulston N., Van Slack A., Mason G. (2012). Negative affective states and their effects on morbidity, mortality and longevity. Anim. Welf..

[13] Hampton J.O., Teh-White K. (2019). Animal welfare, social license, and wildlife use industries. J. Wildl. Manag..

[14] Finn H. (2019). Legal frameworks for wild animal welfare. Aust. Environ. Rev..

[15] Temple D., Manteca X., Velarde A., Dalmau A. (2011). Assessment of animal welfare through behavioural parameters in Iberian pigs in intensive and extensive conditions. Appl. Anim. Behav. Sci..

[16] Samuel E.K., Whay H.R., Mullan S. (2012). A preliminary study investigating the physical welfare and welfare code compliance for tethered and free-ranging horses on common land in South Wales. Anim. Welf..

[17] Andreasen S.N., Sandøe P., Forkman B. (2014). Can animal-based welfare assessment be simplified? A comparison of the Welfare Quality^®^ protocol for dairy cattle and the simpler and less time-consuming protocol developed by the Danish Cattle Federation. Anim. Welf..

[18] Heath C.A.E., Browne W.J., Mullan S., Main D.C.J. (2014). Navigating the iceberg: Reducing the number of parameters within the Welfare Quality^®^ assessment protocol for dairy cows. Animals.

[19] Mullan S., Szmaraged C., Hotchkiss I., Whay H.R. (2014). The welfare of long-line tethered and free-ranging horses kept on public grazing land in South Wales. Anim. Welf..

[20] Dalla Costa E., Dai F., Lebelt D., Scholz P., Barbieri S., Canali E., Zanella A.J., Minero M. (2016). Welfare assessment of horses: The AWIN approach. Anim. Welf..

[21] Blatchford R.A. (2017). Poultry welfare assessments: Current use and limitations. J. Anim. Sci..

[22] Czycholl I., Büttner K., Klingbeil P., Krieter J. (2018). An Indication of Reliability of the Two-Level Approach of the AWIN Welfare Assessment Protocol for Horses. Animals.

[23] Hockenhull J., Whay H.R. (2014). A review of approaches to assessing equine welfare. Equine Vet. Educ..

[24] Hill S.P., Broom D.M. (2009). Measuring zoo animal welfare: Theory and practice. Zoo Biol..

[25] Whitham J.C., Wielebnowski N. (2009). Animal-based welfare monitoring: Using keeper ratings as an assessment tool. Zoo Biol..

[26] Clegg I.L.K., Borger-Turner J.L., Eskelinen H.C. (2015). C-Well: The development of a welfare assessment index for captive bottlenose dolphins (*Tursiops truncatus*). Anim. Welf..

[27] Kagan R., Carter S., Allard S. (2015). A universal animal welfare framework for zoos. J. Appl. Anim. Welf. Sci..

[28] Sherwen S.L., Hemsworth L.M., Beausoleil N.J., Embury A., Mellor D.J. (2018). An animal welfare risk assessment process for zoos. Animals.

[29] Littin K.E., Mellor D.J., Warburton B., Eason C.T. (2004). Animal welfare and ethical issues relevant to the humane control of vertebrate pests. N. Z. Vet. J..

[30] Littin K.E., Fisher P., Beausoleil N.J., Sharp T. (2014). Welfare aspects of vertebrate pest control and culling: Ranking control techniques for humaneness. Rev. Off. Int. Epizoot..

[31] Baker S.E., Ellwood S.A., Watkins R., MacDonald D.W. (2005). Non-lethal control of wildlife: Using chemical repellents as feeding deterrents for the European badger *Meles meles*. J. Appl. Ecol..

[32] Baker S.E., Sharp T.M., Macdonald D.W. (2016). Assessing animal welfare impacts in the management of European rabbits (*Oryctolagus cuniculus*), European moles (*Talpa europaea*) and Carrion crows (*Corvus corone*). PLoS ONE.

[33] Gray M.E., Cameron E.Z. (2010). Does contraceptive treatment in wildlife result in side effects? A review of quantitative and anecdotal evidence. Reproduction.

[34] Sharp T., Saunders G. (2011). A Model for Assessing the Relative Humaness of Pest Animal Control Methods.

[35] Beausoleil N.J., Fisher P., Mellor D.J., Warburton B. (2012). Ranking the negative impacts of wildlife control methods may help advance the Three Rs. ALTEX Proc..

[36] Beausoleil N.J., Mellor D.J. (2015). Advantages and limitations of the Five Domains model for assessing welfare impacts associated with vertebrate pest control. N. Z. Vet. J..

[37] Hampton J.O., Cowled B.D., Perry A.L., Miller C.J., Jones B., Hart Q. (2014). Quantitative analysis of animal-welfare outcomes in helicopter shooting: A case study with feral dromedary camels (*Camelus dromedarius*). Wildl. Res..

[38] Hampton J.O., Forsyth D.M., Mackenzie D., Stuart I. (2015). A simple quantitative method for assessing animal welfare outcomes in terrestrial wildlife shooting: The European rabbit as a case study. Anim. Welf..

[39] Hampton J.O., Hyndman T.H., Barnes A., Collins T. (2015). Is wildlife fertility control always humane?. Animals.

[40] Hampton J.O., Jones B., Perry A.L., Miller C.J., Hart Q. (2016). Integrating animal welfare into wild herbivore management: Lessons from the Australian Feral Camel Management Project. Rangel. J..

[41] Hampton J.O., Forsyth D.M. (2016). An assessment of animal welfare for the culling of peri-urban kangaroos. Wildl. Res..

[42] Hampton J.O., Adams P.J., Forsyth D.M., Cowled B.D., Stuart I.G., Hyndman T.H., Collins T. (2016). Improving animal welfare in wildlife shooting: The importance of projectile energy. Wildl. Soc. Bull..

[43] Hampton J.O., Robertson H., Adams P.J., Hyndman T.H., Collins T. (2016). An animal welfare assessment framework for helicopter darting: A case study with a newly developed method for feral horses. Wildl. Res..

[44] Hampton J.O., Edwards G.P., Cowled B.D., Forsyth D.M., Hyndman T.H., Perry A.L., Miller C.J., Adams P.J., Collins T. (2017). Assessment of animal welfare for helicopter shooting of feral horses. Wildl. Res..

[45] Sharp T.M., McLeod S.R., Leggett K.E.A., Gibson T.J. (2015). Evaluation of a spring-powered captive bolt gun for killing kangaroo pouch young. Wildl. Res..

[46] Allen B.L., Allen L.R., Ballard G., Drouilly M., Fleming P.J.S., Hampton J.O., Hayward M.W., Kerley G.I.H., Meek P.D., Minnie L. (2019). Animal welfare considerations for using large carnivores and guardian dogs as vertebrate biocontrol tools against other animals. Biol. Conserv..

[47] Hing S., Hampton J.O., Gibson T.J. (2019). Animal welfare and the killing of wildlife by captive bolt in Australia. Aust. Zool..

[48] Mellor D.J., Reid C.S.W. (1994). Concepts of Animal Well-Being and Predicting the Impact of Procedures on Experimental Animals. Improving the Well-Being of Animals in the Research Environment.

[49] Mellor D.J., Patterson-Kane E., Stafford K.J. (2009). The Sciences of Animal Welfare.

[50] Mellor D.J., Beausoleil N.J. (2015). Extending the ‘Five Domains’ model for animal welfare assessment to incorporate positive welfare states. Anim. Welf..

[51] Mellor D.J. (2017). Operational details of the five domains model and its key applications to the assessment and management of animal welfare. Animals.

[52] Beausoleil N.J., Mellor D.J., Baker L., Baker S.E., Bellio M., Clarke A.S. (2018). “Feelings and Fitness” Not “Feelings or Fitness”–The *Raison d’être* of Conservation Welfare, Which Aligns Conservation and Animal Welfare Objectives. Front. Vet. Sci..

[53] Green T.C., Mellor D.J. (2011). Extending ideas about animal welfare assessment to include ‘quality of life’ and related concepts. N. Z. Vet. J..

[54] Stafford K.J. (2013). Animal Welfare in New Zealand.

[55] Mellor D.J., Hunt S., Gusset M. (2015). Caring for Wildlife: The World Zoo and Aquarium Animal Welfare Strategy.

[56] Mellor D.J. (2016). Updating animal welfare thinking: Moving beyond the ‘Five Freedoms’ towards ‘A life worth living’. Animals.

[57] Duncan I.J.H. (2006). The changing concept of animal sentience. Appl. Anim. Behav. Sci..

[58] Fraser D. (2008). Understanding Animal Welfare: The Science in It’s Cultural Context.

[59] Broom D.M. (2016). Considering animals’ feeling. Anim. Sentience.

[60] Broom D.M. (2016). Sentience and animal welfare: New thoughts and controversies. Anim. Sent..

[61] Ledger R., Mellor D.J. (2018). Forensic use of the five domains model for assessing animal welfare compromise when preparing expert opinions for animal cruelty prosecutions. Animals.

[62] Mellor D.J. (2019). Welfare-aligned sentience: Enhanced capacities to experience, interact, anticipate, choose and survive. Animals.

[63] Beausoleil N.J., Mellor D.J., Greyling J. (2017). Validating indicators of sheep welfare. Achieving Sustainable Production of Sheep.

[64] Panksepp J. (2005). Affective consciousness: Core emotional feelings in animals and humans. Conscious. Cognit..

[65] Murrell J.C., Johnson C.B. (2006). Neurophysiological techniques to assess pain in animals. J. Vet. Pharmacol. Ther..

[66] Boissy A., Manteuffel G., Jensen M.B., Moe R.O., Spruijt B., Keeling L.J., Winckler C., Forkman B., Dimitrov I., Langbein J. (2007). Assessment of positive emotions in animals to improve their welfare. Physiol. Behav..

[67] Denton D.A., McKinley M.J., Farrell M., Egan G.F. (2009). The role of primordial emotions in the evolutionary origin of consciousness. Conscious. Cognit..

[68] Kenward H., Pelligand L., Savary-Bataille K., Elliott J. (2015). Nausea: Current knowledge of mechanisms, measurement and clinical impact. Vet. J..

[69] Mellor D.J. (2015). Positive animal welfare states and encouraging environment-focused and animal-to-animal interactive behaviours. N. Z. Vet. J..

[70] McMillan F.D. (2003). A world of hurts—Is pain special?. JAVMA.

[71] Gregory N.G. (2004). Physiology and Behaviour of Animal Suffering.

[72] Verbeek E., Waas J.R., McLeay L.M., Matthews L.R. (2011). Measurement of feeding motivation in sheep and the effects of food restriction. Appl. Anim. Behav. Sci..

[73] Verbeek E., Oliver M.H., Waas J.R., McLeay L.M., Blache D., Matthews L.R. (2012). Reduced cortisol and metabolic responses of thin ewes to an acute cold challenge in mid-pregnancy: Implications for animal physiology and welfare. PLoS ONE.

[74] Verbeek E., Waas J.R., Oliver M.H., McLeay L.M., Ferguson D., Matthews L.R. (2012). Motivation to obtain a food reward of pregnant ewes in negative energy balance: Behavioural, metabolic and endocrine considerations. Horm. Behav..

[75] Verbeek E., Ferguson D., Lee C. (2014). Are hungry sheep more pessimistic? The effects of food restriction on cognitive bias and the involvement of ghrelin in its regulation. Physiol. Behav..

[76] Ashley F.H., Waterman-Pearson A.E., Whay H.R. (2005). Behavioural assessment of pain in horses and donkeys: Application to clinical practice and future studies. Equine Vet. J..

[77] Gregory N.G. (1998). Physiological Mechanisms Causing Sickness Behaviour and Suffering in Diseased Animals. Anim. Welf..

[78] Fraser D., Duncan I.J. (1998). ‘Pleasures’, ‘pains’ and animal welfare: Toward a natural history of affect. Anim. Welf..

[79] Spinka M., Newberry R.C., Bekoff M. (2001). Mammalian Play: Training for the Unexpected. Q. Rev. Biol..

[80] Held S.D.E., Špinka M. (2011). Animal play and animal welfare. Anim. Behav..

[81] Yeates J.W., Main D.C.J. (2008). Assessment of positive welfare: A review. Vet. J..

[82] Deag J.M. (1996). Behavioural ecology and the welfare of extensively farmed animals. Appl. Anim. Behav. Sci..

[83] Balcombe J.P. (2009). Animal pleasure and its moral significance. Appl. Anim. Behav. Sci..

[84] Spinka M., Wemelsfelder F., Appleby M.C., Mench J.A., Olsson I.A.S., Hughes B.O. (2011). Environmental challenge and animal agency. Animal Welfare.

[85] Yeates J.W. (2018). Naturalness and Animal Welfare. Animals.

[86] Portas T. (2013). Achieving positive animal welfare outcomes in zoos and aquariums, when coping is not enough: Promoting positive welfare states in animals. Proceedings of the RSPCA Australia Scientific Seminar.

[87] Littlewood K.E., Mellor D.J. (2016). Changes in the Welfare of an Injured Working Farm Dog Assessed Using the Five Domains Model. Animals.

[88] Beausoleil N.J., Fisher P., Littin K.E., Warburton B., Mellor D.J., Dalefield R.R., Cowan P. (2016). A systematic approach to evaluating and ranking the relative animal welfare impacts of wildlife control methods: Poisons used for lethal control of brushtail possums (*Trichosurus vulpecula*) in New Zealand. Wildl. Res..

[89] McGreevy P., Berger J., de Brauwere N., Doherty O., Harrison A., Fiedler J., Jones C., McDonnell S., McLean A., Nakonechny L. (2018). Using the Five Domains Model to Assess the Adverse Impacts of Husbandry, Veterinary, and Equitation Interventions on Horse Welfare. Animals.

[90] Clegg I.L.K., Delfour F. (2018). Can We Assess Marine Mammal Welfare in Captivity and in the Wild? Considering the Example of Bottlenose Dolphins. Aquat. Mamm..

[91] McKenzie R. (2012). Australia’s Poisonous Plants, Fungi and Cyanobacteria: A Guide to Species of Medical and Veterinary Importance.

[92] Dalla Costa E., Murray L., Dai F., Canali E., Minero M. (2014). Equine on-farm welfare assessment: A review of animal-based indicators. Anim. Welf..

[93] Somerville R., Brown A.F., Upjohn M. (2018). A standardised equine-based assessment tool used for six years in low and middle income countries. PLoS ONE.

[94] Henneke D.R., Potter G.D., Kreider J.L., Yeates B.F. (1983). Relationship between condition score, physical measurements and body fat percentage in mares. Equine Vet. J..

[95] Carroll C.L., Huntington P.J. (1988). Body condition scoring and weight estimation of horses. Equine Vet. J..

[96] Gleerup K.B., Forkman B., Lindegaard C., Andersen P.H. (2015). An equine pain face. Vet. Anaesth. Anal..

[97] Dalla Costa E., Minero M., Lebelt D., Stucke D., Canali E., Leach M.C. (2014). Development of the Horse Grimace Scale (HGS) as a Pain Assessment Tool in Horses Undergoing Routine Castration. PLoS ONE.

[98] Hampton J.O., Hyndman T.H., Laurence M., Perry A.L., Adams P., Collins T. (2016). Animal welfare and the use of procedural documents: Limitations and refinement. Wildl. Res..

[99] Linklater W.L., Cameron E.Z., Minot E.O., Stafford K.J. (1999). Stallion harassment and the mating system of horses. Anim. Behav..

[100] Cameron E.Z., Linklater W.L., Stafford K.J., Minot E.O. (2008). Maternal investment results in better foal condition through increased play behaviour in horses. Anim. Behav..

[101] Ransom J.I., Cade B.S. (2009). Quantifying equid behavior: A research ethogram for free-roaming feral horses. U.S. Geological Survey Techniques and Methods Report 2-A9.

[102] Ransom J.I., Cade B.S., Hobbs N.T. (2010). Influences of immunocontraception on time budgets, social behavior, and body condition in feral horses. Appl. Anim. Behav. Sci..

[103] Wemelsfelder F., Hunter E.A., Lawrence A.B., Mendl M.T. (2001). Assessing the ‘whole-animal’: A Free- Choice-Profiling approach. Anim. Behav..

[104] Wemelsfelder F. (2007). How animals communicate quality of life: The qualitative assessment of behaviour. Anim. Welf..

[105] Hintze S., Murphy E., Bachmann I., Wemelsfelder F., Würbel H. (2017). Qualitative Behaviour Assessment of Horses Exposed to Short-term Emotional Treatments. Appl. Anim. Behav. Sci..

[106] Minero M., Dalla Costa E., Dai F., Canali E., Barbieri S., Zanella A., Pascuzzo R., Wemelsfelder F. (2018). Using qualitative behaviour assessment (QBA) to explore the emotional state of horses and its association with human-animal relationship. Appl. Anim. Behav. Sci..

[107] Wemelsfelder F., Hunter E.A., Mendl M.T., Lawrence A.B. (2000). The spontaneous qualitative assessment of behavioural expressions in pigs: First explorations of a novel methodology for integrative animal welfare measurement. Appl. Anim. Behav. Sci..

[108] Keay J.M., Singh J., Gaunt M.C., Kaur T. (2006). Fecal glucocorticoids and their metabolites as indicators of stress in various mammalian species: A literature review. J. Zoo Wildl. Med..

[109] Sherwen S.L., Fanson K. (2015). Validation of an assay to measure glucocorticoid metabolites in the droppings of little penguins (*Eudyptula minor*). J. Zoo Aquar. Res..

[110] Linklater W., Macdonald E., Flamand J.R.B., Czekala N.M. (2010). Declining and low fecal corticoids are associated with distress, not acclimation to stress, during the translocation of African rhinoceros. Anim. Conserv..

[111] Rakotoniaina J.H., Kappeler P.M., Kaesler E., Hämäläinen A.M., Kirschbaum C., Kraus C. (2017). Hair cortisol concentrations correlate negatively with survival in a wild primate population. BMC Ecol..

[112] Kalliokoski O., Jellestad F.K., Murison R. (2019). A systematic review of studies utilizing hair glucocorticoids as a measure of stress suggests the marker is more appropriate for quantifying short-term stressors. Sci. Rep..

[113] Barnard C.J., Hurst J.L. (1996). Welfare by design: The natural selection of welfare criteria. Anim. Welf..

[114] Klingel H. (1975). Social organization and reproduction in equids. J. Reprod. Fertil..

[115] Grange S., Duncan P., Gaillard J.M. (2009). Poor horse traders: Large mammals trade survival for reproduction during the process of feralization. Proc. Biol. Sci..

[116] Garrott R.A., Taylor L. (1990). Dynamics of a feral horse population in Montana. J. Wildl. Manag..

[117] Linklater W.L., Cameron E.Z., Minot E.O., Stafford K.J. (2004). Feral horse demography and population growth in the Kaimanawa Ranges, New Zealand. Wildl. Res..

[118] Harvey A.M., Meggiolaro M.N., Hall E., Watts E.T., Ramp D., Šlapeta J. (2019). Wild horse populations in south-east Australia have a high prevalence of Strongylus vulgaris and may act as a reservoir of infection for domestic horses. Int. J. Parasitol. Parasites Wildl..

[119] Slivinska K., Dvojnos G., Kopij G. (2006). Helminth fauna of sympatric Przwalski’s *Equus przewalskii* Poljav, 1881 and domestic horses *E. caballus* L. in the Chernobyl exclusion zone, Ukraine. Helminthologia.

[120] Klugh D., Klugh D. (2010). Principles of occlusal equilibration. Principles of Equine Dentistry.

[121] Dixon P., du Toit N., Dacre I., Easley J., Dixon P., Schumacher J. (2011). Equine dental Pathology. Equine Dentistry.

[122] Linklater W.L., Cameron E.Z., Stafford K.J., Veltman C.J. (2000). Social and spatial structure and range use by Kaimanawa wild horses (*Equus caballus*: *Equidae*). N. Z. J. Ecol..

[123] Cameron E., Linklater W., Stafford K., Minot E. (2003). Social grouping and maternal behaviour in feral horses (*Equus caballus*): The influence of males on maternal protectiveness. Behav. Ecol. Sociobiol..

[124] Scorolli A.L., Lopez Cazorla A.C. (2010). Demography of feral horses (*Equus caballus*): A long-term study in Tornquist Park, Argentina. Wildl. Res..

[125] Beausoleil N.J., Mellor D.J., Stafford K.J. (2004). Methods for Marking New Zealand Wildlife: Amphibians, Reptiles and Marine Mammals.

[126] Mellor D.J., Beausoleil N.J., Stafford K.J. (2004). Marking Amphibians, Reptiles and Marine Mammals: Animal Welfare, Practicalities and Public Perceptions in New Zealand.

[127] Calvo B., Furness R.W. (1992). A review of the use and the effects of marks and devices on birds. Ring Migrat..

[128] Casper R.M. (2009). Guidelines for the instrumentation of wild birds and mammals. Anim. Behav..

[129] Walker K.A., Trites A.W., Haulena M., Weary D.M. (2012). A review of the effects of different marking and tagging techniques on marine mammals. Wildl. Res..

[130] Hawkins P. (2004). Bio-logging and animal welfare: Practical refinements. Mem. Natl. Inst. Polar Res..

[131] Hampson B.A., de Laat M.A., Mills P.C., Pollitt C.C. (2010). Distances travelled by feral horses in ‘outback’ Australia. Equine Vet. J..

[132] Silver S.C., Ostro L.E.T., Ma L.K. (2004). The use of camera traps for estimating jaguar *Panthera onca* abundance and density using capture/recapture analysis. Oryx.

[133] Ullas Karanth K., Nichols J.D., Samba Kuma N., O’Connell A.F., Nichols J.D., Karanth K.U. (2010). Estimating of Demographic Parameters in a Tiger Population from Long-term Camera Trap Data. Camera Traps in Animal Ecology: Methods and Analyses.

[134] Si X., Kays R., Ding P. (2014). How long is enough to detect terrestrial animals? Estimating the minimum trapping effort on camera traps. PeerJ.

[135] Van Gemert J.C., Verschoor C.R., Mettes P., Epema K., Koh L.P., Wich S., Agapito L., Bronstein M., Rother C. (2014). Nature Conservation Drones for Automatic Localization and Counting of Animals. Computer Vision—ECCV 2014 Workshops.

[136] Vas E., Lescroël A., Duriez O., Boguszewski G., Grémillet D. (2015). Approaching birds with drones: First experiments and ethical guidelines. Biol. Lett..

[137] Ivošević B., Han Y.-G., Cho Y., Kwon O. (2015). Use of conservation drones in ecology and wildlife research. J. Ecol. Environ..

[138] Linklater W.L., Henderson K.M., Cameron E.Z., Stafford K.J., Minot E.O. (2000). The robustness of faecal steroid determination for pregnancy testing Kaimanawa feral mares under field conditions. N. Z. Vet. J..

[139] Sigurjónsdóttir H., Haraldsson H. (2019). Significance of Group Composition for the Welfare of Pastured Horses. Animals.

